# Corrigendum

**DOI:** 10.1111/cns.13292

**Published:** 2020-06-21

In the following article,^1^ the authors would like to correct Figure 4. The correct version of the figure is provided below.

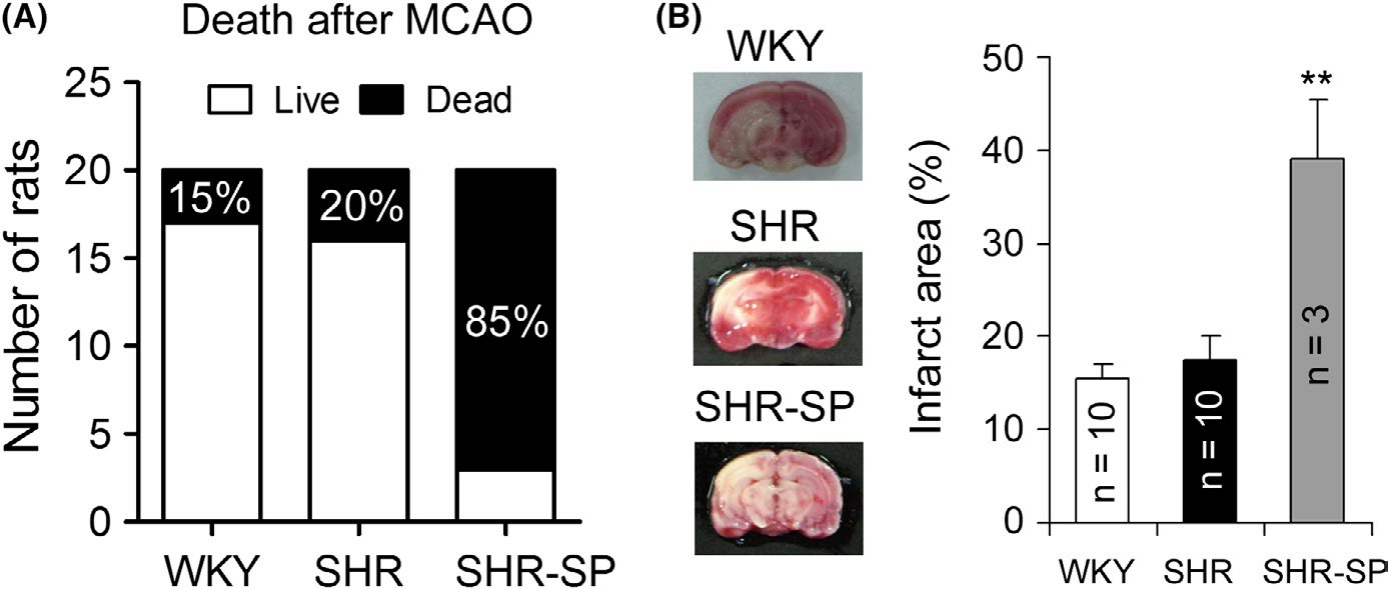

The authors apologize for the error.
